# Gene Editing, Gene Therapy, and Cell Xenotransplantation: Cell Transplantation Across Species

**DOI:** 10.1007/s40472-017-0157-6

**Published:** 2017-07-21

**Authors:** Nizar I. Mourad, Pierre Gianello

**Affiliations:** Pôle de chirurgie expérimentale et transplantation, Université catholique de Louvain, SSS/IREC/CHEX, Avenue Hippocrate, 55 – Bte B1.55.04, 1200 Brussels, Belgium

**Keywords:** Xenotransplantation, Genetic modification, Pig, Pancreatic islets, Diabetes, Neural transplantation

## Abstract

**Purpose of Review:**

Cell xenotransplantation has the potential to provide a safe, ethically acceptable, unlimited source for cell replacement therapies. This review focuses on genetic modification strategies aimed to overcome remaining hurdles standing in the way of clinical porcine islet transplantation and to develop neural cell xenotransplantation.

**Recent Findings:**

In addition to previously described genetic modifications aimed to mitigate hyperacute rejection, instant blood-mediated inflammatory reaction, and cell-mediated rejection, new data showing the possibility of increasing porcine islet insulin secretion by transgenesis is an interesting addition to the array of genetically modified pigs available for xenotransplantation. Moreover, combining multiple modifications is possible today thanks to new, improved genomic editing tools.

**Summary:**

Genetic modification of large animals, pigs in particular, has come a long way during the last decade. These modifications can help minimize immunological and physiological incompatibilities between porcine and human cells, thus allowing for better tolerance and function of xenocells.

## Introduction

Cell replacement therapies represent innovative alternative treatments offering the promise of long-lasting restoration or amelioration of disrupted cellular functions rather than temporary alleviation of clinical symptoms necessitating life-long medication. Patients suffering from degenerative and auto-immune diseases could benefit from such treatments. In particular, pancreatic islet allotransplantation for the treatment of type I diabetes became a clinical reality thanks to great advances in islet isolation and preservation and to less aggressive immunosuppression. Transplantation of other cell types such as hepatocytes, bone marrow, or umbilical cord stem cells is also used in the clinic today while neural cell transplantation which could represent a significant step forward in the treatment of neurodegenerative disorders is still in an early development stage. However, and regardless of tissue-specific considerations and difficulties, a common major obstacle standing in the way of wide-spread use of cell transplantation is the lack of human donors, therefore opening the door for the use of alternative sources of cells from other species. In this context, pigs emerged as suitable candidates for providing xenocells due to anatomical and physiological similarities with humans. The possibility of genetically modifying donor pigs to mitigate the host immune reaction to xenografted cells and to adapt their function to human physiology when needed will definitely accelerate the transition of cell xenotransplantation from the bench to the clinic.

## Porcine Pancreatic Islet Xenotransplantation

The rationale behind using porcine pancreatic islets to treat type I diabetes in humans stems from the similarity between porcine and human insulin and the possibility to isolate large amounts of islets from a reasonable number of donors [1]. The diabetic non-human primate model has been used extensively to show the efficacy of transplanted adult and neonatal porcine islets with variable degrees of success in a number of preclinical studies as reviewed here [1–3]. Naturally, preserving implanted islets from the host immune response was central in most of these studies. Once this primordial step has been achieved by means of immunosuppression, encapsulation, genetic modification of donors, or a combination of these strategies, the efficacy of porcine islet transplantation is evaluated by looking for signs of diabetes alleviation and by verifying secretory function of grafted tissue. Moreover, host testing for possible porcine pathogens is undertaken to demonstrate biosafety of islet xenotransplantation. With regard to all of these factors that will determine the outcome of pig islet transplantation in preclinical and clinical trials, genetic modification of donors is set to become an unavoidable step.

### Modification of Donor Pigs to Improve Insulin Secretion

**Fig. 1:**
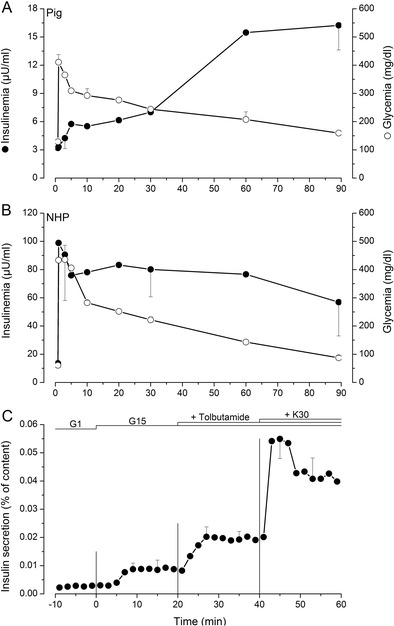
In vivo and in vitro insulin secretion from porcine islets. **a**, **b** Glucose (○) and insulin (●) were measured in plasma after intravenous glucose challenge (0.5 mg/kg) in piglets (**a**) and non-human primates (*NHP*; **b**). **c** In vitro insulin secretion from perifused piglet islets exposed to 1 mM glucose (*G1*) then stimulated with 15 mM glucose (*G15*). Potassium channel blocker, tolbutamide (500 μM), then 30 mM KCl (*K30*) were added to the perifusion medium as indicated on top of the figure. Values are means ± SEM from *n* = 3–5 intravenous glucose tolerance tests (*IVGTTs*) and *n* = 4 different preparations for islet perifusions

**Fig. 2:**
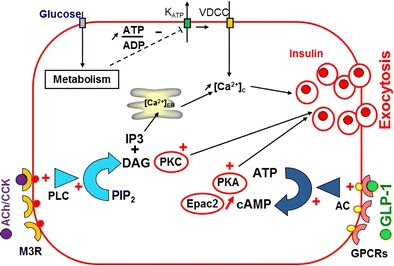
Triggering and amplifying mechanisms of insulin secretion in pancreatic beta cells. Glucose metabolism increases the ATP/ADP ratio in beta cells. This leads to closure of ATP-dependent potassium channels (*K*_*ATP*_) and depolarization of the plasma membrane which in turn causes opening of voltage-dependent calcium channels (*VDCC*), thus allowing entry of calcium and increase of cytosolic calcium concentration (*[Ca* ^*2+*^ *]c*). This rise in [Ca^2+^]c triggers insulin granule exocytosis. Insulin secretion can be further amplified by glucagon-like peptide 1 (*GLP-1*) binding to its G-protein-coupled receptor (*GPCR*) and activation of adenylyl cyclase (*AC*) which converts ATP to cAMP. The rise of cytosolic cAMP then activates protein kinase A (*PKA*) and exchange protein activated by cAMP (*Epac2*). Activation of a type-3 muscarinic receptor (*M3R*) by acetylcholine (*ACh*) or cholecystokinin (*CCK*) activates phospholipase C (*PLC*) which hydrolyzes phosphatidylinositol-4, 5-bisphosphate (*PIP*_*2*_) into the protein kinase C activator (*PKC*) diacylglycerol (*DAG*) and inositol trisphosphate (*IP3*) which mobilizes intracellular calcium stores

Although porcine insulin differs from its human counterpart by a single amino acid and despite similar islet insulin contents in adult porcine and human islets (616 and 725 μU/IEQ, respectively), there exist considerable differences between the two with regard to their cell composition and most importantly their physiology. Porcine islets contain fewer α-cells (8%) compared to human islets (30%) [4] and most of these cells are further lost during isolation due to their peripheral localization within porcine islets. From a functional point of view, porcine islets respond to glucose stimulation with a 2–3-fold increase of insulin secretion whereas human islets show a 10–12-fold increase in response to a similar stimulation [5•]. This particularity of porcine beta cells probably explains the need to transplant a large number of islets (up to 50,000 IEQ/kg receiver body weight) [6, 7] for the graft to produce physiologically relevant amounts of insulin. We and others [3, 8•] believe that neonatal pigs represent the most realistic source of islets for xenotransplantation in a clinical setting. Neonatal pig islets are technically and logistically easier to isolate compared to islets from adult pigs. However, these islets are not completely matured and their insulin content and secretion are lower than their adult counterparts [5•]. On the other hand, they have been shown to undergo further maturation in vitro (personal observation) and in vivo after transplantation [9]. Surprisingly, there has been very little effort made to study porcine islet physiology in order to understand the mechanisms behind this low secretory activity of porcine islets both in vivo and in vitro. During an intravenous glucose tolerance test (IVGTT), pig glycemia quickly peaks 1–3 min following glucose injection then gradually returns to pre-stimulatory levels over a period of 90 min (Fig. 1a). Plasma insulin measurement over the same period shows a delayed lingering increase that continues up to the 90-min mark (Fig. 1a). In comparison, there is a sevenfold increase of insulinemia within 3 min of glucose injection in macaques (Fig. 1b). In vitro perifusion experiments corroborate in vivo data as 15 mM glucose only causes a threefold increase of insulin secretion. As shown in Fig. 1c, porcine islets respond to stimulation with a sulfonylurea (tolbutamide) and secretion is further increased upon depolarization with 30 mM KCl indicating functional stimulus-secretion coupling in their beta cells. Key cell membrane receptors involved in these pathways such as sulfonylurea receptor (SUR1) and glucagon-like peptide-1 receptor (GLP1R) were shown to be expressed in pig islets [10]. In a recently published study, we showed that genetic modification of pig beta cells can be targeted to increase glucose-stimulated insulin secretion by activating key pathways known to regulate insulin secretion in mouse [11, 12] and human [13] islets. Transgenic expression of a DPPV (dipeptidyl peptidase V)-resistant form of GLP-1 (glucagon-like peptide-1) and of a constitutively activated form of a type-3 muscarinic receptor (M3R) using an adenoviral vector allowed a significant fourfold amplification of glucose-stimulated insulin secretion from both adult and neonatal perifused pig islets [5•]. Pathways and effectors involved in the effects of GLP-1 and muscarinic activation on beta cells are identified and mainly depend on cAMP-dependent activation of protein kinase A (PKA) and exchange protein directly activated by cAMP (Epac2) as well as a rise of cytosolic calcium and protein kinase C (PKC) activation (Fig. 2) as described previously [14, 15]. By genetically modifying these two pathways, we could obtain porcine islets that secrete as much insulin as human islets at least in vitro [5•]. Transgenic local production of GLP-1 within mouse islet cells in vitro has been previously described. This has been achieved by means of adenoviral-mediated expression of GLP-1 [16] or of prohormone convertase (PC) 1/3 which cleaves GLP-1 from proglucagon in α-cells [17]. In both cases, increased GLP-1 production in islet cells was accompanied by increased insulin secretion, enhanced survival in response to cytokine treatment, and protection against H_2_O_2_-induced stress. Transplantation of GLP-1-expressing mouse islets under the kidney capsule of syngeneic diabetic mice allowed a return to normoglycemia in 88% of treated animals compared to 52% of animals that had received unmodified islets [18]. Transgenic mice expressing activated M3R at the β-cell level have been produced [19]. These mice exhibited improved glucose tolerance and increased serum insulin levels. In vitro, islets isolated from these mice showed greater insulin secretion compared to wild-type islets. Inducing these genetic modifications in a novel pig model will be targeted to pancreatic beta cells by adding a porcine insulin promoter to the construct used to transfect primary fibroblasts. In addition, targeting the expression cassette towards a safe-harbor site within the pig genome using specific transcription activator-like effector nuclease (TALEN) pairs should permit to obtain a single integration and stable expression of the desired transgenes.

### Modification of Donor Pigs to Mitigate the Immune Response

Immediately after transplantation to human or non-human primate recipients, porcine islets are subjected to hyperacute rejection due to anti-αGal (galactose α 1,3 galactose) preformed antibodies binding to the αGal oligosaccharide epitope expressed on porcine cells followed by complement activation and neutrophil infiltration. This humoral reaction is even stronger against islets from neonatal pigs since they show higher expression of the αGal epitope compared to adult pigs [20]. A major breakthrough in this field was the knockout of both alleles of the α 1,3-galactosyltransferase enzyme (GGTA1) which synthesizes the αGal epitope resulting in the first transgenic pigs lacking this major xenoantigen [21]. Compared to wild-type islets, GGTA1-KO neonatal islets showed superior secretory function after hepatic transplantation to diabetic immunosuppressed macaques and were less susceptible to complement-mediated destruction in vitro [7]. More recently, double knockout of GGTA1 and of CMAH (cytidine monophosphate-*N*-acetylneuraminic hydroxylase) responsible for Neu5Gc (*N*-glycolylneuraminic acid) synthesis was shown to further decrease porcine islet antigenicity compared to GGTA1 knockout alone [22]. A recent paper by Salama et al. showed that such a double knockout in porcine beta cells is possible without affecting islet function both in vivo and in vitro [23]. Another porcine carbohydrate antigen produced by beta-1, 4-*N-*acetyl-galactosaminyl transferase 2 (B4GALNT2) was identified by screening cDNA from GGTA1^−/−^ pig endothelial cells with sera from primates that had rejected GGTA1^−/−^ pig hearts [24, 25]. Expression of B4GALNT2 in human cells significantly increased antibody-dependent complement-mediated lysis when these cells were challenged with serum from pig-to-baboon cardiac xenotransplantation sensitized recipient serum [25] while its deletion in pig PBMCs efficiently reduced human IgG and IgM binding and decreased human anti-porcine cytotoxicity [26, 27]. The fact that a strong humoral response was detected in non-human primates receiving alginate-encapsulated islets especially against αGal [28, 29] further emphasizes the necessity to use GGTA1-KO pigs as a background for other genetic modifications. Instant blood-mediated inflammatory reaction (IBMIR) is initiated upon contact between host blood and xenografted tissue. This innate non-specific reaction characterized by thrombin generation and complement activation [30] is caused by tissue factor expression on islets cells [31] and further aggravated by anti-pig antibodies. Encapsulated islets implanted in poorly vascularized sites might escape this reaction at least in the early stages of engraftment but IBMIR causes a quick loss of up to 60% of islets implanted in the portal vein [32]. Transgenic expression of human CD46 [33•, 34], CD55, and CD59 [35] and knockdown of tissue factor gene [36] were shown to avoid this early damage to the graft by decreasing IBMIR both in vivo and in vitro. hCD46 expression in porcine islets increased graft survival to more than 3 months compared to only 46 days in the case of wild-type islets transplanted to immunosuppressed macaques [34]. Transgenic neonatal porcine islets expressing CD55 and CD59 on a GGTA1-KO background were less susceptible to humoral injury in vitro and did not cause intraportal thrombosis in baboons but suffered from cell-mediated rejection [35]. The impact of tissue factor knockdown in neonatal porcine islets on IBMIR was demonstrated in vitro by treating islets with tissue factor-specific antisense RNA and exposing them to human blood. Reduced clot formation, complement, and coagulation activation were observed with modified islets compared to control islets [36]. In mice, expression of human endothelial protein C receptor (hEPCR) on islets improved graft survival and function, reduced inflammation and coagulation, and allowed diabetes correction using less islets than required when wild-type mice were used as donors [37]. Anti-apoptotic and anti-inflammatory molecules heme oxygenase-1 (HO-1) and A20 were shown to be efficient in preserving transgenic porcine endothelial cells against TNF-α-induced apoptosis [38, 39]. In islets, expression of HO-1 prolonged pig-to-mouse graft survival and decreased immune cell infiltration and islet cell apoptosis [40]. In the same study, the authors showed that human soluble tumor necrosis factor-α receptor-Fc (sTNF-αR-Fc) expression produced a similar effect but did not protect islet cells against apoptosis during early engraftment period. However, neither sTNF-αR-Fc nor HO-1 suppressed anti-pig humoral reaction as attested by anti-pig IgG and IgM levels 1 month post-implantation [40]. Expression of human leukocyte antigen-E (HLA-E) on porcine endothelial cells decreased natural killer cell-mediated cytotoxicity in vitro and prolonged survival time of pig lungs perfused with human blood ex vivo [41]. Perhaps, the most challenging immunological hurdle to overcome in xenotransplantation is the adaptive immune system’s cell-mediated response. Indeed, in most cases, islets that survive early damage due to hyperacute rejection and IBMIR are eventually destroyed by T-cell and macrophage infiltration of the graft [42, 43, 44•]. To ensure long-term survival of transplanted islets in non-human primates, severe immunosuppression is necessary [45, 46]. Indeed, without immunosuppression, neonatal porcine islets were destroyed within 4 days following transplantation and this was accompanied by T-cell and macrophage infiltration of the graft site. A CD28-CD154 co-stimulation blockade regimen allowed longer survival of implanted islets and diabetic recipients remained insulin-independent up to 260 days [45]. Comparable results were obtained using adult porcine islets with graft function observed up to 187 days in macaques treated with CD25- and CD154-specific antibodies together with a pretransplant induction therapy using basiliximab and a combination of three drugs for immunosuppression maintenance [46]. However, such aggressive immunosuppressive regimens are not applicable in humans. A much more elegant approach would be to engineer islet cells to produce immunosuppressive antibodies or molecules locally at the graft site, thus preserving the islets and sparing the host from complications of systemic immunosuppression. Pig islets expressing anti-human CD2 [47], porcine cytotoxic T lymphocyte-associated antigen (pCTLA4) [48], or co-stimulatory signal inhibitor LEA29Y [49] have been described. Whereas transgenic pCTLA4 expression in pigs seemed to compromise their immune system [48], beta cell-specific or ubiquitous LEA29Y expression had no deleterious effect on pig health or reproduction [49, 50]. Pig-to-mouse transplantation of anti-CD2- or LEA29-expressing islets benefited from CD3^+^ T-cell depletion and protection against human peripheral blood mononuclear cells respectively [47, 49] but pig-to-primate transplantation of pCTLA4-expressing islets did not provide significant amelioration of long-term survival (less than 5 months) [51]. Indeed, comparable survival time was reported in non-human primates implanted with wild-type encapsulated islets without immunosuppression [29]. More recently, microencapsulated neonatal porcine islets were shown to survive and maintain function over a maximal period of 600 days in diabetic patients [8•] suggesting that immunoisolation by cell encapsulation might be a better alternative to prevent cell-mediated rejection and permit long-term survival of transplanted pig islets [52].

### Modification of Donor Pigs to Decrease the Risk of Zoonosis

Among porcine microorganisms, porcine endogenous retroviruses (PERVs) are of particular concern as they are encoded within pig DNA and therefore cannot be eliminated by pathogen-free breeding. So far, no evidence for PERV infection in human porcine islet recipients has been reported [53, 54]. However, as in vitro infection of human tumor cell lines and even primary cells has been documented [55, 56], current recommendations call for selection of donors with low levels of genomic PERV copies and low PERV expression [53, 57]. In this context, attempts to reduce or suppress PERV expression by genetic modification of donors have been made. Small interfering RNAs have been successfully used to generate transgenic pigs with decreased PERV expression compared to wild-type animals [58, 59] and this inhibitory effect lasted up to 3 years [60]. Zinc finger nuclease-driven knockout of PERV proviral sequences proved to be difficult to achieve since it was accompanied by severe cytotoxicity possibly due to the high number of PERV inserts in the genome [61]. Gene editing using clustered regularly interspaced short palindromic repeat-associated system CRISPR/Cas9 successfully inactivated 62 genomic copies of PERV in a porcine kidney cell line [62•] suggesting that generation of completely PERV-free pigs might be possible. In a recently published study, we analyzed PERV expression in an array of pig tissues with particular emphasis on pancreatic islets [63•]. Although we found PERV expression to be enriched in islet cells and therefore comparable to other non-immune tissues, we could not detect PERV particles or RT activity in islet culture media. This data together with the lack of evidence for PERV infection in human receivers of live porcine tissues might call for reconsideration of the actual risk caused by PERV in the case of islet xenotransplantation although it might be of greater relevance for other tissues.

## Neural Xenotransplantation and Gene Therapy

Neural cell xenotransplantation is an attractive alternative therapy for neurodegenerative diseases affecting neural tissues devoid of complex and precise neuron connections such as the nigrostriatal dopamine neurons involved in Parkinson’s disease (PD) [64]. In PD, the aim of cell transplantation is to restore dopamine production in the striatum and reverse impairments in motor behavior. Fetal human dopamine neurons were shown to survive and function up to 10 years following transplantation in PD patients [65, 66]. In a pioneer phase I clinical study, fetal porcine ventral mesencephalic and striatal cells were implanted to patients with PD or Huntington’s disease [67]. Despite 30% of treated patients showing some improvement, very few surviving porcine cells were found in one deceased patient autopsied 7 months post-implantation. Despite immunological privileges of the brain, T-cell-mediated immune rejection of grafted tissue is a major concern in neural cell xenotransplantation [68] and cyclosporine A immunosuppression was not sufficient to protect neural cell xenografts in rats [69, 70]. As an alternative to immunosuppression, transgenic expression of human CTLA4 in porcine neurons inhibited human T lymphocytes proliferation in vitro without affecting normal development after transplantation in rats [71]. Genetic modifications can also enhance transplanted cells’ function or even confer new functions. In a primate model of PD, gene therapy by lentiviral expression of glial cell line-derived neurotrophic factor (GDNF) resulted in significant GNDF expression for up to 8 months and reversed motor deficits [72]. Overexpression of VEGF and GNDF in transplanted human umbilical cord blood mononuclear cells (UCB-MCs) successfully improved symptoms of spinal cord injury in rats [73]. As with other porcine tissues, pig neural cell transplantation carries the risk of PERV transmission as discussed earlier. However, no PERV infection was detected in patients transplanted with neural fetal pig cells [74].

## Conclusion

Pancreatic islet xenotransplantation is the most investigated cell xenotransplantation application in both research and clinical settings. This field has benefited from improvement of islet isolation techniques, cell encapsulation, availability of reliable experimental models, and better understanding of risks inherent to pig cell use in humans. Recent advances in genome editing tools allowed production of multi-transgenic pigs with efficient expression of desired genes [41, 51, 75, 76•, 77] without causing deleterious effects on islet function [23, 33•]. Most if not all of these genetic modifications of donors are aimed to mitigate immune rejection of grafted cells without the need for radical immunosuppression. Physiological incompatibilities between porcine and human islets need to be taken into consideration and genetic modification of pig beta cells aimed to increase insulin secretion are being investigated with promising results [5•]. Combining these genetic engineering strategies with microbiological selection of animals and pathogen-free breeding as well as cell encapsulation techniques is bringing therapeutic islet xenotransplantation closer than ever to widespread application.
